# Combining optogenetics with sensitive FRET imaging to monitor local microtubule manipulations

**DOI:** 10.1038/s41598-020-62874-3

**Published:** 2020-04-07

**Authors:** Orry Van Geel, Stephanie Cheung, Theodorus W. J. Gadella

**Affiliations:** 1 Swammerdam Institute for Life Sciences, Section of Molecular Cytology, van Leeuwenhoek Centre for Advanced Microscopy, University of Amsterdam, Science Park 904, 1098XH Amsterdam, The Netherlands; 2 Present Address: Developmental Biology Unit, European Molecular Biology Laboratory, 69117 Heidelberg, Germany

**Keywords:** Confocal microscopy, Optogenetics, Fluorescent proteins, Microtubules

## Abstract

Optogenetic methods for switching molecular states in cells are increasingly prominent tools in life sciences. Förster Resonance Energy Transfer (FRET)-based sensors can provide quantitative and sensitive readouts of altered cellular biochemistry, e.g. from optogenetics. However, most of the light-inducible domains respond to the same wavelength as is required for excitation of popular CFP/YFP-based FRET pairs, rendering the techniques incompatible with each other. In order to overcome this limitation, we red-shifted an existing CFP/YFP-based OP18 FRET sensor (COPY) by employing an sYFP2 donor and mScarlet-I acceptor. Their favorable quantum yield and brightness result in a red-shifted FRET pair with an optimized dynamic range, which could be further enhanced by an R125I point mutation that stimulates intramolecular interactions. The new sensor was named ROPY and it visualizes the interaction between the microtubule regulator stathmin/OP18 and free tubulin heterodimers. We show that through phosphorylation of the ROPY sensor, its tubulin sequestering ability can be locally regulated by photo-activatable Rac1 (PARac1), independent of the FRET readout. Together, ROPY and PARac1 provide spatiotemporal control over free tubulin levels. ROPY/PARac1-based optogenetic regulation of free tubulin levels allowed us to demonstrate that depletion of free tubulin prevents the formation of pioneer microtubules, while local upregulation of tubulin concentration allows localized microtubule extensions to support the lamellipodia.

## Introduction

The field of optogenetics is continually expanding, producing a steady stream of new light responsive tools and applications with an increasing impact on science^1–3^. Most of the optogenetic tools contain protein domains that perform their function in response to light from the blue end of the spectrum, e.g. LOV domains, channelrhodopsins, cryptochromes, etc.^1,4^. Unfortunately, the same part of the spectrum is used for another popular technique in microscopy as well, namely Förster resonance energy transfer (FRET). Most FRET sensors are developed with cyan and yellow fluorescent protein (FP) pairs since they have good characteristics for FRET imaging based on donor quenching and acceptor sensitization^5,6^. In order to combine blue-sensitive optogenetics with FRET imaging, more red-shifted fluorophores are needed to avoid crosstalk from overlapping excitation windows. The donor needs to be excited above 514 nm, which rules out the use of cyan FPs. Yellow FPs like mVenus, SYFP2, mCitrine or orange FPs like mKO or mOrange fulfill this requirement. For these donors, red FPs or far red FPs are required as acceptor, respectively. However, most (far) red fluorescent proteins are compromised with low quantum yields and/or maturation problems, hampering robust and efficient red-shifted FRET sensors. Recently, improved red FPs have been developed that allow for novel and efficient FRET sensing with red-shifted spectral properties^7^. We utilized these improved red FPs for creating a suitable red-shifted FRET pair that can be efficiently excited at 514 nm^7,8^. To this end we incorporated new red-shifted FRET pairs into the COPY sensor, which originally had a YFP/CFP pair attached to the N- and C-terminus of a stathmin/oncoprotein18 (OP18) molecule^9^. Stathmin is an important negative regulator of microtubule dynamics^10,11^, and will shift the dynamic instability of microtubules towards depolymerization when it captures free tubulin dimers in a 2:1 ratio^12^. This tubulin sequestering function is believed to be the driving force behind the stimulation of microtubule catastrophes^10^. Although the N-terminal part is known to interact with microtubule tips as well, which can potentially stimulate depolymerization to some extent^13,14^. There are four serine residues that can become phosphorylated in stathmin via a number of cellular signaling pathways, among which is the Rac1-PAK1 pathway^15–17^. Phosphorylation diminishes stathmin’s affinity for tubulin and its tertiary structure shifts from a large elongated α-helix to an unstructured chain^18^. Stathmin’s control over tubulin availability is critical in a number of cellular processes like mitosis^9,19^, and helps to tightly regulate the balance of microtubule dynamics in cooperation with the other microtubule regulatory factors^20^. So far, the effect of stathmin has only been studied through stimulation of entire cells^9,13,21^, whereas it is known that microtubule dynamics are typically regulated locally. In this study we aimed to generate a new optogenetic application with FRET readout to allow simultaneous local control of microtubule dynamics and monitoring of local free tubulin concentrations. To this end we created a novel red-shifted stathmin sensor we termed ROPY. We show the compatibility of the ROPY FRET sensor with blue light responsive optogenetics and demonstrate that localized release of tubulin can stimulate microtubule growth in that area.

## Results

### Generating a red-shifted FRET sensor with suitable dynamic range

**Figure 1:**
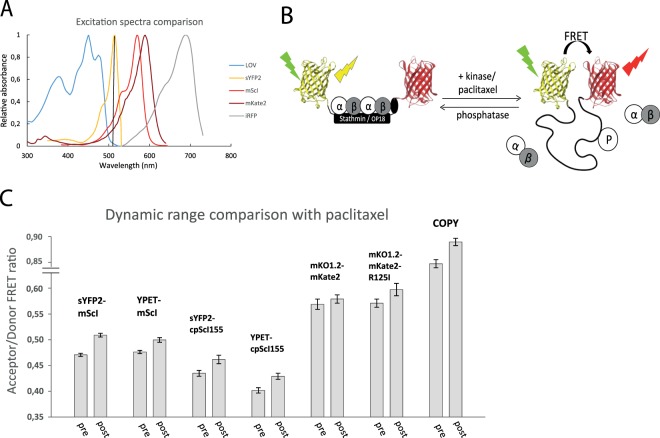
Generating a red-shifted FRET sensor with suitable dynamic range. (**A**) The excitation spectrum of a common blue-responsive optogenetics domain is plotted together with the fluorescent proteins used in this study to indicate the minimal amount of spectral overlap present. (**B**) Schematic overview of how the stathmin FRET-sensor cycles between elongated and unstructured states depending on tubulin binding. (**C**) Ratiometric FRET comparison of several FP combinations we tested, before and after addition of 10 µM paclitaxel. The sensors were transiently expressed in HEK293T cells and averages for each condition were calculated from n > 400 cells. Error bars indicate the 95% confidence intervals.

The original CFP/YFP-based COPY sensor is not suitable for experiments with blue light responsive optogenetics due to the impossibility to excite the donor without activating the optogenetic module. We aimed to simultaneously manipulate and monitor the free tubulin concentration by using an asLOV2-based optogenetic switch^22^ in combination with FRET. Hence, we needed to red-shift the fluorescent proteins used in the COPY sensor to ones that can be excited above 500 nm (Fig. 1A). The COPY sensor reports on the difference between the monomeric disordered stathmin state where the terminal FPs can interact, and the stabilized elongated α-helix through the binding of tubulin where the terminal FPs are too far apart to interact^9,18^. Such changes in FRET can be induced by altering stathmin’s tubulin-binding properties through phosphorylation or by modulating the intracellular free tubulin concentration, for instance with a drug like paclitaxel which prevents microtubule depolymerization^21^ (Fig. 1B). In a first attempt we combined orange fluorescent protein donors with near-infrared fluorescent protein acceptors, yet we could not find a combination with a dynamic range above background noise (Fig. 1C). This is partly due to the sensor’s design displaying an intrinsically low dynamic range, and in part related to poor quantum yield and maturation problems of the employed acceptor FP probes. We therefore decided to focus on FP pairs which can elicit the maximum theoretical FRET potential in the desired excitation range, i.e. a donor FP with high quantum yield with optimal excitation at 514 nm and acceptor with high brightness and efficient maturation^6,8^. mScarlet-I (mScI) was chosen as the best FRET acceptor currently available in the red spectrum^7^ and we combined it with yellow FPs either Ypet^23^ or sYFP2^24^. The excitation spectra show that activation of YFP at 514 nm can theoretically still slightly trigger the edge of the LOV spectrum as well (Fig. 1A), however in our experience this did not activate the LOV domain in any detectable way if the laser intensity was kept low. We compared ratiometric FRET changes of both wildtype and circular permutated versions of the red-shifted sensor upon adding 10 µM of paclitaxel for 1 h that reduces the free tubulin concentration and hence enhances FRET (Fig. 1C). Altering the FP orientation with circular permutation did not improve the FRET interaction and it became clear that a sYFP2-mScI pair had the largest FRET response out of all the explored combinations (Table 1). This FRET pair allows one additional orthogonal detection channel in the near-infrared part of the spectrum that can be used for localizing proteins of interest tagged by a near-infrared fluorescent probe, e.g. iRFP713 (Fig. 1A).

**Table 1:** Overview of relative FRET ratio changes upon paclitaxel stimulation.

Table 1	Relative FRET increase
sYFP2-mScI	1,081 ± 0,015
YPET-mScI	1,049 ± 0,016
YPET-cpSc155	1,068 ± 0,028
sYFP2-cpSc155	1,062 ± 0,032
mKO1.2-mKate2	1,017 ± 0,031
mKO1.2-mKate2-R125I	1,046 ± 0,035
COPY	1,065 ± 0,024
ROPY-4A	1,146 ± 0,021
ROPY	1,113 ± 0,022
ROPY-4E	1,014 ± 0,016
ROPY-Y67Gs	0,998 ± 0,025
ROPY (DMSO)	1,007 ± 0,018

Values were obtained by subtracting the normalized average FRET ratios before and after paclitaxel addition, represented with ±95% confidence interval of n > 400 for each condition.

### Optimization and characterization of the ROPY FRET sensor

**Figure 2:**
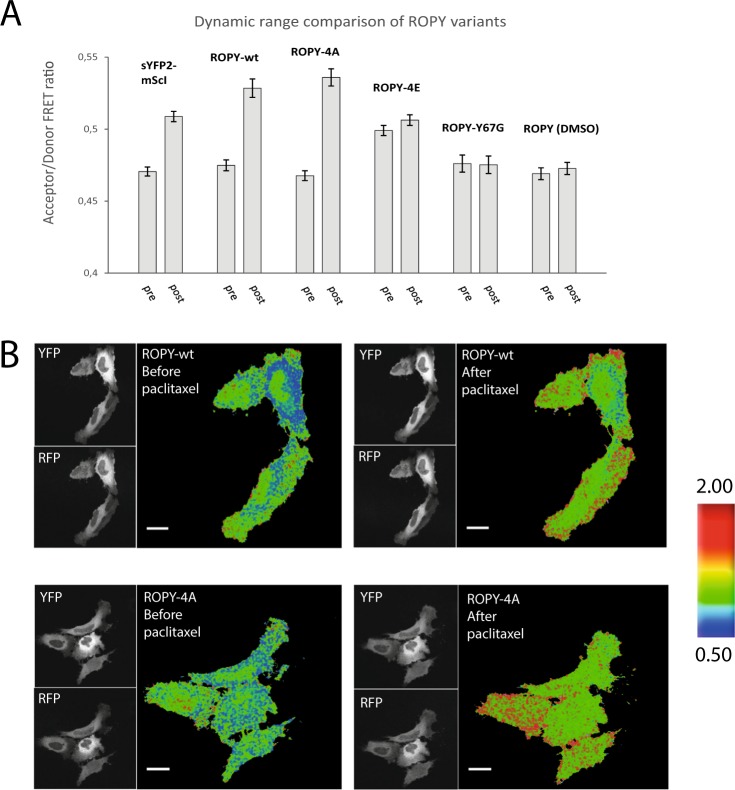
Optimization and characterization of new ROPY FRET sensor. (**A**) RFP/YFP ratiometric FRET comparison of several ROPY mutations, before and after addition of 10 µM paclitaxel, one control condition used DMSO without paclitaxel. The sensors were transiently expressed in HEK293T cells and averages for each condition were calculated from n > 600 cells. Error bars indicate the 95% confidence intervals. (**B**) Confocal images of YFP and RFP emissions along with their RFP/YFP ratio for ROPY variants before and after 5 minutes of 10 µM paclitaxel addition. Experiment was performed in HeLa cells. Scale bars represent 20 µm.

Once we established the optimal sYFP2-mScI FRET pair for the stathmin sensor, we looked into modifying its properties in order to improve the dynamic range. Since the FRET interaction of the sensor is dependent on the proximity of both FPs in the tubulin unbound state, we attempted to reverse the monomeric mutation in coral derived FPs by introducing a R125I point mutation^25^. The mutation successfully increased the dynamic range in the coral derived mKO1.2-mKate2 pair (Table 1), yet this pair was sub-optimal, so we also tried if the R125I mutation would have the same effect in mScarlet-I. Indeed, R125I successfully increased the dynamic range for mScI as well (Fig. 2A), and we named this final optimized version ROPY (RFP-OP18-YFP). Next, we were interested in determining whether the R125I mutation induced the higher FRET response through mScarlet homo-dimerization or through sYFP2-mScI hetero-dimerization. To this end, we introduced a Y67G point mutation into either sYFP2 or mScI which abolishes the formation of the FP-chromophore in either FP. Upon expressing a mix of ROPY sensors with chromophore-less sYFP2 and mScI proteins (Fig. S1), we found no FRET response after addition of paclitaxel (Fig. 2A), which suggests that the increased FP interaction due to the R125I mutation is not intermolecular but intramolecular in nature. The fact that the baseline FRET ratios are similar further dismisses intermolecular interactions.

We evaluated the properties of the ROPY sensor further by making a phospho-mimetic variant with four serines mutated into glutamic acids (ROPY-4E) and a phospho-blocking variant with four serines mutated into alanines (ROPY-4A), as previously reported in the literature^9,26^. Respectively, these mutants should minimize and maximize the sensor’s affinity for tubulin, thereby indicating the lower and upper range of the ROPY sensor signal. Indeed, when we compared ratiometric FRET changes upon adding 10 µM of paclitaxel for 1 h, we detected hardly any difference for ROPY-4E and a bigger difference for ROPY-4A as compared to the wildtype (Fig. 2A). This confirms that the regulatory tubulin sequestering ability of ROPY is still functioning like wildtype stathmin^9,16^.

On a single cell scale, it becomes clear that the wildtype stathmin sensor has a more inhomogeneous spatial distribution of the FRET response than ROPY-4A (Fig. 2B), which was detected with the original COPY sensor as well^9^. This suggests that endogenous phosphorylation of the sensor occurs mostly near the cell edges.

Interestingly, we observed a rising variability in the baseline FRET ratios with increasing ectopically expressed ROPY sensor concentrations (Fig. S2). Also the difference in FRET readout between ROPY-4E and ROPY-4A is greatly diminished at higher sensor concentrations (not shown), which could sometimes be a result of bystander FRET at elevated sensor concentrations. However, based on the negative result for intermolecular FRET we think it is more likely that in case of overexpression of the sensor, ROPY will titrate out the available free tubulin in the cell causing an increasingly lower tubulin occupancy of the sensor and thereby a reduction of FRET and sensitivity of the sensor.

### Combining ROPY with blue responsive optogenetics

**Figure 3:**
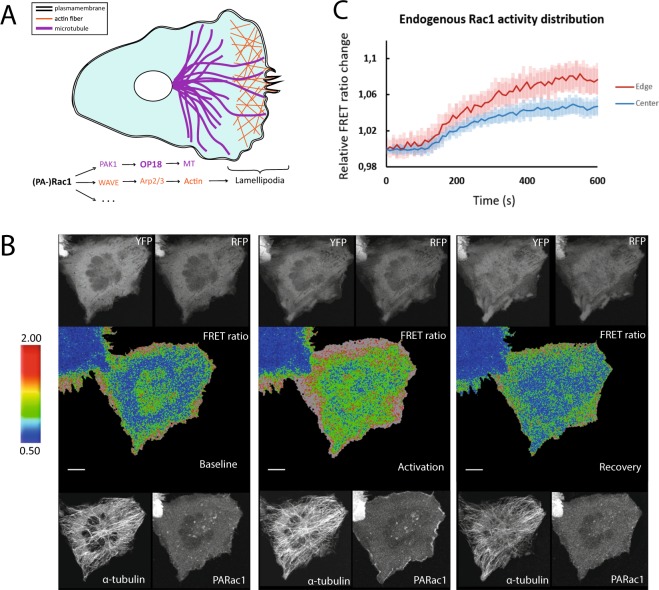
Combining ROPY with blue responsive optogenetics. (**A**) Schematic overview of the signaling pathways affected by Rac1 which result in cytoskeletal growth and lamellipodia formation. (**B**) Confocal images of ROPY, PARac1 & α-tubulin-iRFP co-expression in HeLa cells. Baseline was acquired in the dark state, the activation images were taken 5 minutes after illuminating the entire cell with 458 nm pulses every 20 s, and recovery images were taken 5 minutes after the last 458 nm light pulse was given. Scale bars represent 10 µm (**C**) Normalized ratiometric ROPY changes over time in response to PARac1 activation. The FRET signal in HeLa cells was divided in an outer region of 5 µm thickness (red) and the remaining inner region (blue). Error bars represent the standard deviation, n = 10.

The next step was to test the compatibility of the new red-shifted ROPY sensor with a blue light responsive optogenetic construct, PARac1. The Rac1 Rho-GTPase affects many signaling pathways involved in cytoskeletal organization^27^ (Fig. 3A), including OP18 regulation through phosphorylation, which disrupts tubulin-sequestering by OP-18 (Fig. 1B). The spectral characteristics of ROPY allow for an additional fluorescent tag to be imaged in the far-red spectrum (Fig. 1A), so we co-expressed ROPY, PARac1 with an mTq2 fluorescent tag, and α-tubulin with an iRFP713 tag^28^ (Fig. 3B). All channels except for CFP could be independently imaged while no response from PARac1 was detected during the baseline. The mTq2 tag could only be imaged during the activation step starting right after the final baseline image of the ROPY channels was taken, but this is enough to also detect the increase in PARac1 concentration at the plasma membrane during its activation. We illuminated the whole cell with 458 nm light during 5 minutes, followed by 5 minutes recovery time in the dark. During this time we observed the FRET ratio increase in the entire cell followed by a decrease back to baseline levels. The FRET-increase implies PARac1-induced phosphorylation of OP-18, releasing tubulin from OP-18 and allowing a closer proximity of the SYFP2 donor to the mScI acceptor in the ROPY sensor (Fig. 1B). The subcellular inhomogeneous distribution of ROPY activity is present throughout all timepoints to varying degrees. Not only is the spatial FRET gradient usually highest near the edge of the cell, the largest increase in ratio over time also occurs at the edge (Fig. 3C). On average, the changes near the edge of the cell were almost twice as large as the ones in the center. In order to confirm that the observed changes were due to the interplay between ROPY & PARac1, we measured negative controls where either the sensor could not be phosphorylated (ROPY-4A) or where Rac1 could not be activated (PARac1-C450A) and indeed we found no significant responses under those conditions (Fig. S3). This indicates that the changes seen in Fig. 3B reflect phosphorylation of the ROPY sensor through PARac1 optogenetic stimulation.

### Locally affecting the tubulin affinity of ROPY

**Figure 4:**
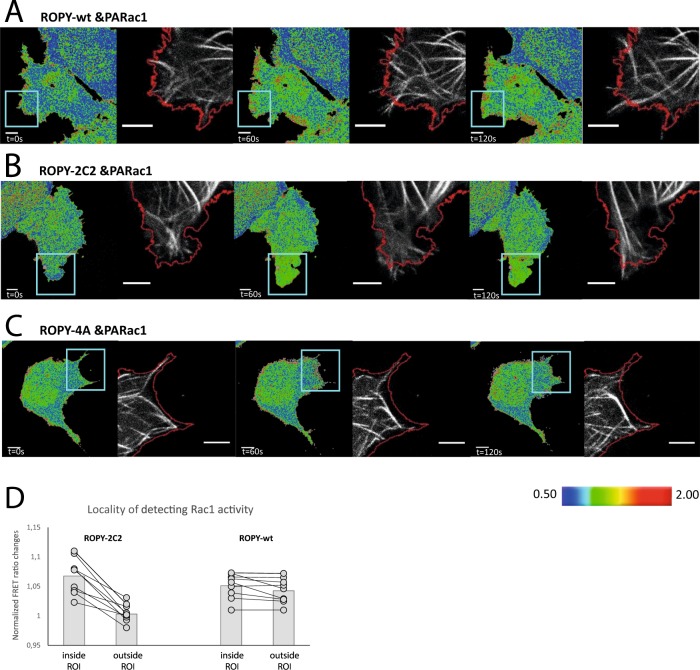
Locally affecting the tubulin affinity of ROPY. (**A–C**) Confocal images of ROPY variants with PARac1 & EB3-iRFP expressed in HEK293T cells. (**A**) wt free diffusible ROPY with PARac1, (**B**) membrane bound ROPY-2C2 with PARac1 and (**C**) phosphorylation-deficient ROPY-4A with PARac1. The cells were locally illuminated with 458 nm pulses every 20 s within the ROI as indicated in blue. Subsequent microtubule changes are visualized in relation to the outline of the cell at t = 0, indicated in red. The images are representative examples of n > 10 observations. Scale bars represent 6 µm (**D**) HEK293T cells were co-transfected with PARac1 and either ROPY or ROPY-2C2, and locally illuminated with 458 nm for 5 minutes. Normalized ratiometric FRET responses from within the activated ROI are plotted against the changes outside of that ROI, n = 10.

The advantage of optogenetic constructs is that they can be locally activated, thus we examined the response of ROPY to a localized phosphorylation stimulus (Fig. 4A). PARac1 induced membrane ruffling and protrusions, which were most prominent in the area of 458 nm illumination. This is explained through its modulatory effects on the actin cytoskeleton^27,29^. In contrast to the regional membrane ruffling following optogenetic Rac1 stimulation, we found that the ROPY sensor responds similarly to localized or non-localized stimuli, with a FRET increase throughout the entire cell and a gradient from the cell edge to the center (Fig. 3A). This suggests that in the sequence of events leading to the phosphorylation-induced FRET response, certain components apparently display a diffusion rate that is high enough for the phosphorylated sensor to dominate the entire cellular response. This precludes that the combination of PARac1 and ROPY can be used to investigate how locally released tubulin affects the microtubule dynamics in combination with a clear readout of the area where tubulin was released through the sensor. We previously tackled the problem of maintaining spatial resolution with optogenetics^30^, and sought to circumvent this problem in a similar way. We attempted to lower the diffusion of ROPY by adding two lactadherin-C2 lipid binding domains at the C-terminus (ROPY-2C2) that will keep it mostly bound to the plasma membrane^31^. Unlike free diffusing ROPY (Fig. 4A), the ROPY-2C2 version was able to indicate local activation gradients (Fig. 4B). As a control we paired ROPY-4A with PARac1 and detected localized membrane protrusions, indicating Rac1 activation, without a change in stathmin activity as expected (Fig. 4C). We show the disparity of local PARac1 activity detection between free ROPY and ROPY-2C2 for a handful of cells to indicate the need for this membrane bound version (Fig. 4D).

Now that we established a localized release of free tubulin under optogenetic control with FRET readout, we investigated how a localized increase in free tubulin would influence microtubule growth. As a way of visually confirming that tubulin is indeed being released upon stathmin phosphorylation, we locally activated PARac1 in cells expressing ROPY-2C2 and α-tubulin-iRFP. Indeed, we observed that colocalization of these proteins diminished within the area of illumination (Fig. S4). We studied the effect that locally releasing tubulin has on microtubules by expressing iRFP-tagged end-binding protein 3 (EB3) which has a high affinity for growing microtubule +TIPs^32^. We were able to detect microtubules beyond the original cell outline and into the PARac1 induced protrusions, shaping the form of the resulting lamellipodia (Fig. 4A,B), though not for cells expressing ROPY-4A (Fig. 4C). This implies that the microtubule extensions are a direct result of the local release of free tubulin dimers. These microtubule extensions correlate nicely with the areas of increased FRET activity. Protrusions outside of the illuminated region where ROPY-2C2 detected no FRET response did not exhibit this local microtubule growth effect (Fig. S5), indicating that most of the released tubulin is quickly incorporated into local microtubules.

## Discussion

The applications for optogenetics are continuously increasing, however, not many supporting quantitative imaging tools are currently available for the technique. With FRET sensors for instance, the major issue is the spectral overlap of the excitation spectrum of most existing sensors with the activation spectrum of blue light responsive optogenetic domains, which renders both techniques incompatible with each other. Some have stated that single FP-based reporters might be the future of optogenetic readouts^33^, however there are far less of these reporters currently available than FRET sensors, and most single FP-based sensors provide qualitative rather than quantitative readout. Thus at the moment the easiest way of obtaining a quantitative reporter that is compatible with blue responsive optogenetics is to alter the spectral properties of existing FRET probes. Recently, a few attempts have been made to solve this problem, e.g. adding bioluminescent protein donors to existing FRET sensors which rely on coelenterazine additives^34^, or replacing the FPs with a new infrared FP pair^35^. However, these methods have certain practical disadvantages, as bioluminescence requires additives and is accompanied by low photon fluxes and the latter utilizes FPs in the far-red spectrum which to date still have sub-optimal properties for FRET^5,35^, both hampering FRET sensing of subtle effects. Therefore, we decided to create a red-shifted FRET sensor with FPs in the 500 nm + excitation that exhibit high quantum yield and brightness in order to make the FRET pair compatible for sensors with lower dynamic ranges as well^7,8^. The original COPY sensor displayed a relatively low dynamic range, and our failed attempts at maintaining a functional sensor with orange and near-infrared FPs made it clear that an optimized FRET pair with the right spectral properties and a good acceptor FP is really required for low dynamic range sensors. Upon further screening, we identified the combination of sYFP2 as donor with mScI as acceptor to work well. We think that other yellow donors such as mVenus and mCitrine^36,37^, not tested here will probably show similar results. Possibly, the higher quantum yield mNeongreen and mClover3^38,39^ could be used as well, but we decided not to use these FPs as donors in view of their blue shift of ~10 nm as compared to sYFP2 which reduces the spectral overlap with the mScI acceptor and reduces the extinction coefficient at 514 nm with roughly 30–50%. In view of its efficient maturation, high brightness and long Förster radius for FRET from an sYFP2 donor (6.24 nm), mScI turned out to be the key element of the red-shifted ROPY sensor. The R125I mutation that reintroduces a dimerizing surface on the FP^25^ was able to enhance the FRET contrast even further. Remarkably, we determined that this mutation did not stimulate intermolecular dimerization but that it increased only intramolecular FRET, probably by improving the binding affinity between the donor and acceptor FP of tubulin-free stathmin. The biological properties of the sensor were found to be unaltered by the red-shift. Its activity still depends on the change in tubulin affinity through phosphorylation, basal activity was highest at edge of the cells, and a decrease of tubulin incorporation into microtubules that scales with stathmin concentration was also observed, all just as reported for the original COPY sensor^9^. High concentrations of stathmin appeared to disrupt the basal FRET ratio (Fig. S2). As a result of these concentration dependent effects we recommend to use the sensor only at low expression levels for accurate measurements and minimal modulation of the free tubulin concentration.

ROPY could be accurately measured independent of a PARac1 optogenetic construct, though the dynamic range was slightly lower and more variable as compared to paclitaxel stimulation. This is likely due to the basal activity of PARac1 in the dark state at varying concentrations, and the fact that phosphorylation is continuously being reversed in the cell by phosphatases while paclitaxel circumvents this pathway. Dark state activities are always present in optogenetic constructs^1,22^, meaning this can always affect a FRET sensor’s dynamic range paired with it. That is another reason why a FRET pair with optimized efficiency is preferred for optogenetic experiments. PARac1 induced the largest FRET change near the plasma membrane, which makes sense as Rac1 is known to be associated with the plasma membrane when it is activated^40^. Rac1 activation is most frequently discussed in the framework of the resulting lamellipodia and filopodia, which are attributed to downstream actin polymerization^29,41^, though other components like the Rac1-PAK1 pathway can also clearly influence the resulting protrusions as we observed. When we looked at microtubule dynamics in response to a local induction of Rac1 activity, we detected microtubules extending into protrusions, which help to define their shape by acting as a rigid support structure. This response was only present in combination with the wildtype stathmin sensor, and not ROPY-4A, meaning the effect of stathmin and local tubulin availability is important when it comes to cell morphology and motility. It has previously been established that Rac1 activity is required to induce pioneer microtubules that occur at the leading cell edge^11^, and our data suggests that the downstream regulatory mechanisms to achieve this could be as simple as regulating localized tubulin concentrations through stathmin for example. The fact that microtubules extending into protrusions occurred only within the targeted area means that tubulin dimers will quickly be incorporated in nearby microtubules when released, thus allowing a simple capture-and-release function to guide the cytoskeleton. The combination of PARac1 and ROPY-2C2 FRET sensing could be a great tool to further study the cytoskeletal effects of local free tubulin gradients by gaining spatio-temporal control over local tubulin concentrations with a sensory readout. The availability of the near-infrared part of the spectrum with this combination is highly valuable since it allows direct visualization of the elicited response.

By red-shifting the COPY sensor, we showed that it is possible convert existing FRET probes with low dynamic range into optogenetic-compatible ones. The sYFP2 and mScI-R125I fluorescent proteins are a great pair that allow for complete separation of control over blue light responsive protein domains and sensor readouts, while maintaining a solid dynamic FRET range. This approach adds to the possibilities of red-shifting sensors and will hopefully result in more studies combining FRET with optogenetically controlled signalling, since molecular information on spatio-temporally regulated systems could become an invaluable asset in future research.

## Materials & Methods

### Plasmid constructs

The original COPY sensor plasmid was a kind gift from Philippe Bastiaens. The protein coding part was first inserted into a pTriEx-3 plasmid backbone through AgeI and NotI restriction sites for easier cloning and expression purposes. Subsequent exchange of fluorescent proteins (FPs) was achieved by extracting the desired donor FPs from pClontech-FP-C1 plasmids with AgeI and BglII restriction sites. The acceptor FPs were amplified from pClontech-FP-C1 plasmids with forward primer 5′-TGATGGATCCGGTTCA(18xN) and reverse primer 5′GATACCTGAGGGCGGCCGCTCATTT where 18xN represents the start of the specific protein sequences. The amplified domains were then inserted into the sensor plasmid through BamHI and Bsu36I restriction sites.

Generation of the phosphomimic (OP18-4E) and phosphoblock (OP18-4A) mutants of the stathmin sensor was achieved by introducing point mutations through the following primers: S16A/E: forward 5′-TGGAGAAGCGCGCT(GCA/GAA)GGCCAGGCTTTTG and reverse 5′-CAAAAGCCTGGCCTTCAGCGCGCTTCTCCA; S25A/E: forward 5′-GAGCTGATTCTC(GCC/GAA)CCTCGGTCAAAAG and reverse 5′-CTTTTGACCGAGGTTCGAGAATCAGCTC; S38A/E: forward 5′-CGATTTCCCCCTT(GCT/GAG)CCCCCAAAGAAG and reverse 5′-CTTCTTTGGGGGCTCAAGGGGGAAATCG; S63A/E: forward 5′-GAAAGACGCAAG(GCT/GAA)CATGAGGCGGAAG and reverse 5′-CTTCCGCCTCATGTTCCTTGCGTCTTTC.

The R123I mutation in mScI was introduced through amplification with forward primer 5′-CAAGGTGAAGCTCATCGGCACCAACTTCCCTCC and reverse primer 5′-TAGATCAGGGTGCCGTCCTCCAGG. The Y67G mutation was introduced into sYFP2 through amplification with forward primer 5′-CCTCGTGACCACCCTGGGCGGCGGCGTGCAGTGCTTCG and reverse primer 5′-GTGGGCCAGGGCACGGGCAGCTTG, and the same mutation introduced into mScI with forward primer 5′-GTCCCCTCAGTTCATGGGCGGCTCCAGGGCCTTC and reverse primer 5′-AGGATGTCCCAGGAGAAGGGCAG.

The membrane-anchored version of ROPY was made by extracting the sensor domains with AgeI and SgrAI restriction sites for ligation into our previously published 2xC2 plasmid which is available through addgene (114416).

PARac1 and α-tubulin-iRFP713 were obtained from addgene (22027 & 56277) (Cambridge, MA, USA).

We obtained EB3-GFP as a kind gift from Niels Galjart (Rotterdam, Erasmus MC), and EB3-iRFP was subsequently cloned by swapping the iRFP from α-tubulin-iRFP713 through AgeI and BsrGI restriction sites.

All relevant new generated constructs from this publication are available for use through addgene (ID 128002–128009).

### Cell culture & transfection

Human embryonic kidney cells (Hek293, American Type Culture Collection crl-1573) and human HeLa cervical cancer cells (ccl-2) were cultured in Dulbecco’s modified Eagle medium (DMEM) supplemented with Glutamax and 10% fetal bovine serum at 37 °C and 7% CO_2_. All cell culture media were obtained from Invitrogen (Breda, NL). For experiments, all cells were cultured on 25 mm glass coverslips (Menzel- Gläser, Braunschweig, Germany) and transiently transfected with plasmids using 1 μg/ml polyethylenimine approximately 24 h prior to imaging. Hek293 cells were serum starved for 4 h before experiments. Right before imaging the culture medium was replaced with microscopy medium (137 mM NaCL, 5.4 mM KCl, 1.8 mM CaCl2, 0.8 mM MgCl2, 20 mM glucose, and 20 mM HEPES at PH = 7.4).

### Widefield ratiometric FRET measurements

Hek293T cells were imaged on a widefield Zeiss Axiovert 200 M microscope (Carl Zeiss MicroImaging, Jena, Germany) equipped with a 10x Plan Neofluar objective (NA 0.03 air) and a cooled charge-coupled device camera (Hamamatsu ORCA II). Cells were excited using a mecury lamp, at 500/20 nm with a 535/30 nm emission filter for YFP donor signal and a 620/60 nm emission filter for RFP sensitized emission. For OFP donor signals, 500/20 nm excitation was paired with a 572/28 nm emission filter, while the near-infrared sensitized emission was excited with 500/20 nm and detected by a 620/60 nm emission filter. Regular acceptor intensities were obtained by 577/20 excitation with a 620/60 nm emission filter. Cells were stimulated with a 1:1000 dilution of 10 mM paclitaxel stock dissolved in DMSO (Sigma-Aldrich) and imaged again 1 h after incubation. Background and flat-field corrections were applied to all images. Cells with RFP intensity counts above 200 were not analyzed in order to avoid artefacts in the FRET signal due to concentration. The RFP/YFP FRET ratio for each condition was obtained by calculating the slope of plotting Σ(donor × SE)/Σ(donor^2^) for n > 400. The error bars represent the 95% confidence intervals. Measurements were taken from distinct samples.

### Confocal laser scanning microscopy

Experiments were performed on an inverted Leica TCS SP8 microscope (Leica Microsystems, Germany) equipped with a 63 × HC Plan Apo objective (NA 1.4 oil) and HyD hybrid detectors at 37 °C. YFP donor signals were obtained by exciting cells with a 514 nm argon ion laser and detection with a 525–550 nm emission bandpass. RFP sensitized emission signals were obtained by exciting cells with a 514 nm argon ion laser and detection with a 590–625 nm emission bandpass. iRFP signals were obtained by exciting cells with a 633 nm helium-neon laser and detection with a 670–800 nm emission bandpass.

Optogenetic activation was achieved by scanning an ROI of the desired size with a 458 nm argon ion laser line at 40 μW per pixel, repeated every 20 s.

Images were analyzed using ImageJ (National Institutes of Health, Bethesda, MD) for background corrections, calculating RFP/YFP ratio images (Figs. 2C, 3A and 4A), creating overlays of cell outlines (Fig. 4A), and separating a 5 µm thick outer circumference of cells from the remaining core (Fig. 3B). Measurements were taken from distinct samples.

## Supplementary information

Supplementary Information.

Total PA-Rac1 activation with ROPY and EB3.

Local PA-Rac1 activation with ROPY and EB3.

Local PA-Rac1 activation with ROPY-2C2 and EB3.

Local PA-Rac1 activation with ROPY-4A and EB3.

## Data Availability

The underlying datasets from figures in this publication are available from the corresponding author on reasonable request.
