# Supplementary material for: Combining optogenetics with sensitive FRET imaging to monitor local microtubule manipulations
Source: Sci Rep. 2020 Apr 7;10:6034. doi: 10.1038/s41598-020-62874-3 (PMC7138840; doi:10.1038/s41598-020-62874-3)
Supplement: Supplementary file 1 — Supplementary Information. [file 41598_2020_62874_MOESM1_ESM.pdf]

Supplemental Materials

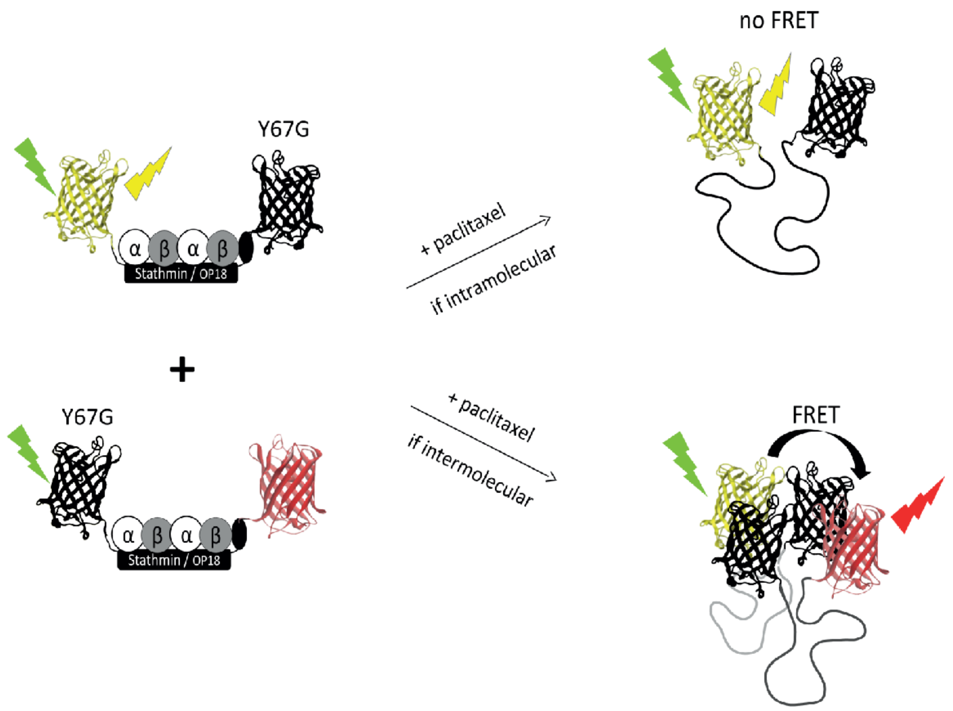

Fig S1. Assessment of altered FP binding affinities due to the R125I point mutation. Schematic overview of the experiment performed in fig 2A, depicting both possible outcomes. We introduced a Y67G point mutation into either FP in order to create inactive versions of ROPY with one non-fluorescent FP-protein structure and one functional sYFP2 or mScI. By themselves these ROPY mutants cannot undergo a FRET interaction upon release of tubulin. However, if the dimerizing R125I mutation introduced into mScI would have a much higher affinity for itself than for differently constructed FP ß-barrels, it could stimulate intermolecular binding which would allow for an intermolecular FRET interaction to occur. Since no FRET was detected during the experiment in fig 2A, we have to assume that the R125I mutation increased ROPY's dynamic range mostly by promoting intramolecular FP interactions.

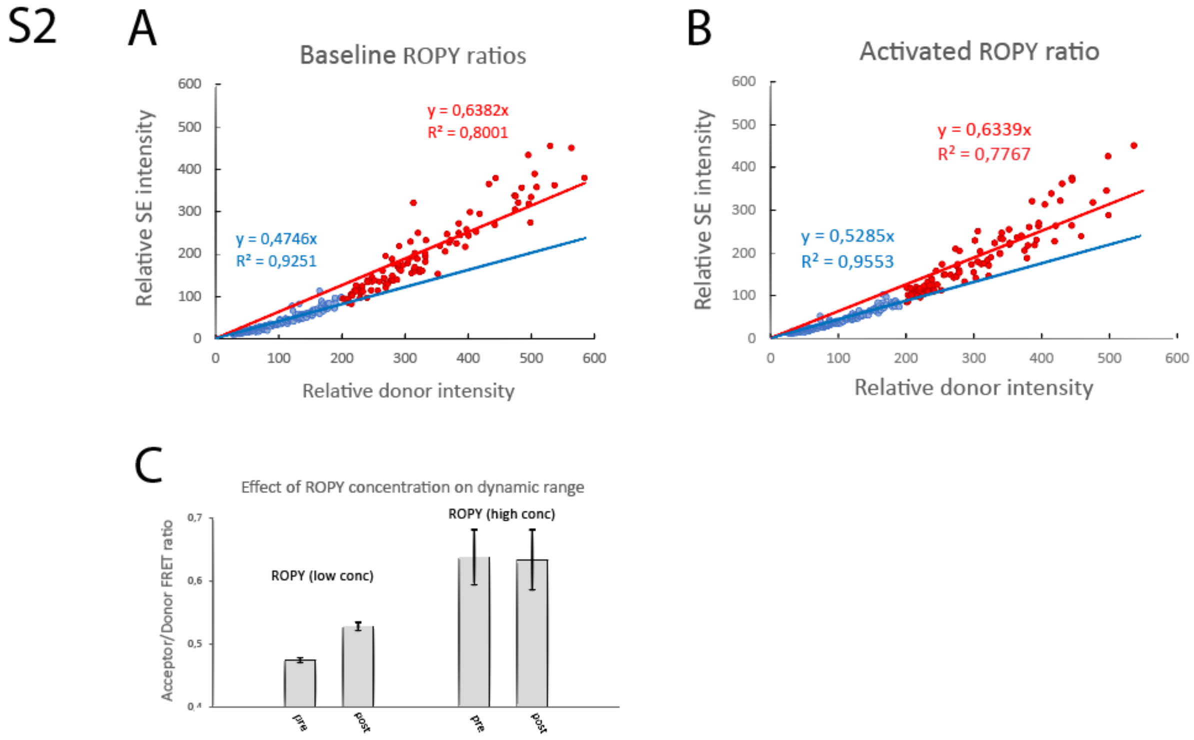

Fig S2. Concentration dependence of ROPY's FRET ratio. (A-B) Individual donor and sensitized emission intensities of basal ROPY (A) and paclitaxel stimulated ROPY (B) are plotted to indicate the effect of sensor concentration on the FRET dynamic range. We arbitrarily set a threshold at a relative donor intensity of 200 since on average the points start to deviate from a linear correlation above this threshold. Cells used in the main analysis are represented in blue and excluded ones in red. The average ratio for each fraction was determined by the slope of their respective trendlines, which has a large error for red points due to the wide spread of FRET ratios at these concentrations. This variation likely reflects the variability in free tubulin concentrations between those cells, which can alter the 2:1 binding ratio with stathmin and disrupt the basal non-FRET state. (C) RFP/YFP ratiometric FRET comparison of high and low ROPY expression levels, both before and after addition of 10µM paclitaxel. Values were obtained from the trendlines in A-B. The ROPY sensor was transiently expressed in HEK293T. Error bars indicate the 95% confidence intervals.

S3

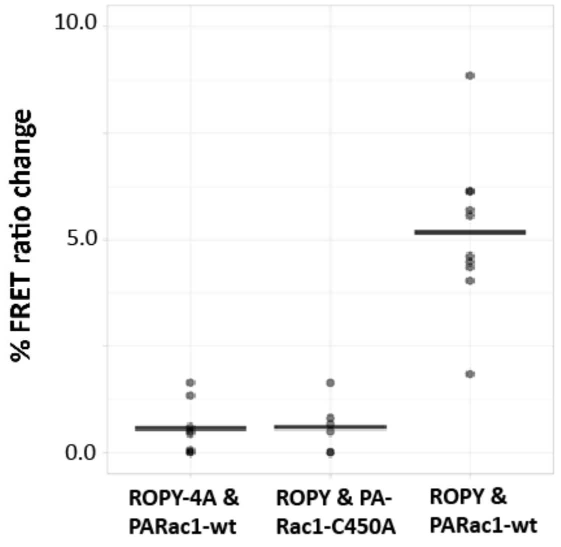

Fig S3. Effect of PA-Rac1 on stathmin. Relative FRET ratio increase of ROPY & PARac1-wt, compared to ROPY & inactive PARac1-C450A, as well as inactive ROPY-4A & PARac1-wt. Without either a phosphorylatable stathmin domain or an active Rac1, no significant FRET change was observed. This confirms that PA-Rac1 is able to indirectly regulate the tubulin binding activity of stathmin. Experiment was performed in HeLa cells under 5 minutes exposure to 458nm light, n=8.

S4

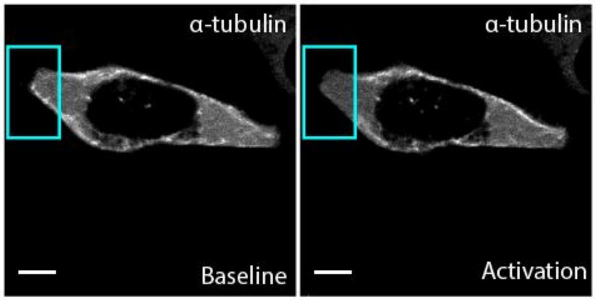

Fig S4. Visualization of ROPY-2C2's tubulin binding ability. In fig 4B we showed that confining ROPY to the plasma membrane resulted in a more local FRET response which correlated nicely with the area where we induced local PA-Rac1 activation. Here we wanted to visually confirm that this more precise spatial representation of Rac1 activity was due to local phosphorylation of stathmin, which is why we co-expressed PA-Rac1, ROPY-2C2, and α-tubulin-iRFP713. We found that local PA-Rac1 activation with 458nm within the blue ROI, trigged the release of bound tubulin within that ROI but not elsewhere, which confirms that ROPY-2C2 can be used as an indicator of locally released tubulin dimers. Experiment was performed with HEK293T cells.

S5

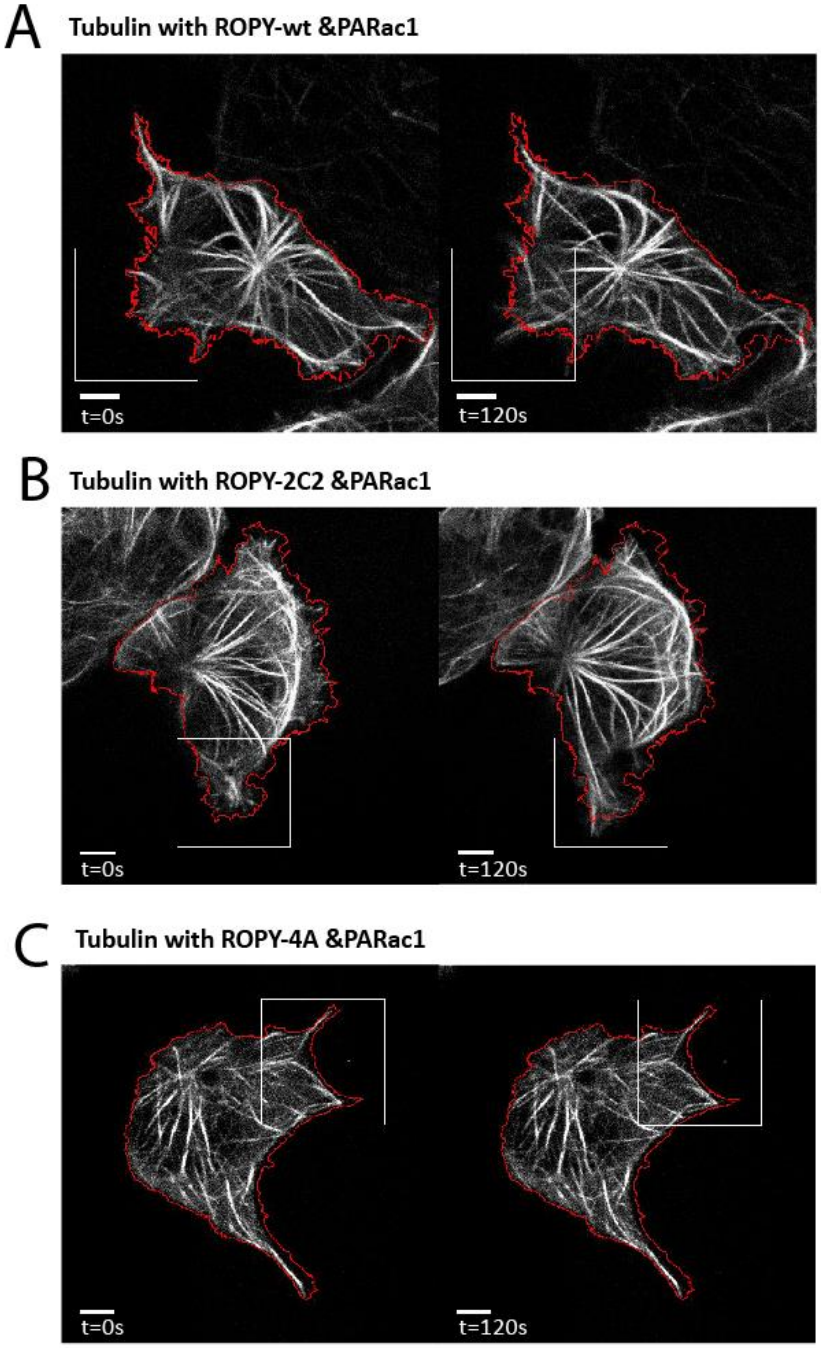

Fig S5. Visualization of ROPY-2C2's tubulin binding ability. (A-C) Whole cell confocal images of EB3-iRFP expressed in HEK293T cells together with A) wt free diffusible ROPY & PA-Rac1 B) membrane bound ROPY-2C2 & PA-Rac1 and C) phosphorylation-deficient ROPY-4A & PA-Rac1. These images are the same as in fig 4A-C except that here the microtubule composition of the entire cells are visible. These enlarged images clearly show that microtubule extensions into the lamellipodia only occurred within the area of PA-Rac1 activation and subsequent tubulin release. The fact that any protrusions outside the ROI don't show microtubule extensions suggests that the released tubuluin will quickly become incorporated into nearby growing microtubules. The cells were locally illuminated with 458nm pulses every 20s within the ROI as indicated. For reference, the outline of the cell at t=0 is indicated in red. The images are representative examples of n>10 observations. Scale bars represent 6 µm.

Captions for movies

Movie S1

HeLa cells co-expressing PA-Rac1, ROPY, and EB3-iRFP713 are stimulated by 458nm light in the entire area. The ROPY FRET ratio and microtubule response are represented side by side. Scale bar represents 10 µm.

Movie S2

A HeLa cell co-expressing PA-Rac1, ROPY, and EB3-iRFP713 is locally stimulated by 458nm light within the indicated ROI. The microtubule response is represented in orange on top of the grey YFP signal from ROPY in order to show the presence of growing microtubules inside the local induced protrusions. Scale bar represents 10 µm.

Movie S3

A HeLa cell co-expressing PA-Rac1, ROPY-2C2, and EB3-iRFP713 is locally stimulated by 458nm light within the indicated ROI. The microtubule response is represented in orange on top of the grey YFP signal from ROPY in order to show the presence of growing microtubules inside the local induced protrusion. Scale bar represents 10 µm.

Movie S4

A HeLa cell co-expressing PA-Rac1, ROPY-4A, and EB3-iRFP713 is locally stimulated by 458nm light within the indicated ROI. The microtubule response is represented in orange on top of the grey YFP signal from ROPY in order to show that no growing microtubules are present inside the local induced protrusions. Scale bar represents 10 µm.
